# Curcumin and resveratrol inhibit nuclear factor-kappaB-mediated cytokine expression in adipocytes

**DOI:** 10.1186/1743-7075-5-17

**Published:** 2008-06-12

**Authors:** Amanda M Gonzales, Robert A Orlando

**Affiliations:** 1 Department of Biochemistry and Molecular Biology, University of New Mexico, School of Medicine, MSC08 4670, 1 University of New Mexico, Albuquerque, New Mexico, 87131, USA

## Abstract

**Background:**

Adipocytes express inflammatory mediators that contribute to the low-level, chronic inflammation found in obese subjects and have been linked to the onset of cardiovascular disorders and insulin resistance associated with type 2 diabetes mellitus. A reduction in inflammatory gene expression in adipocytes would be expected to reverse this low-level, inflammatory state and improve cardiovascular function and insulin sensitivity. The natural products, curcumin and resveratrol, are established anti-inflammatory compounds that mediate their effects by inhibiting activation of NF-κB signaling. In the present study, we examined if these natural products can inhibit NF-κB activation in adipocytes and in doing so reduce cytokine expression.

**Methods:**

Cytokine (TNF-α, IL-1β, IL-6) and COX-2 gene expression in 3T3-L1-derived adipocytes was measured by quantitative real-time PCR (qRT-PCR) with or without TNFα-stimulation. Cytokine protein and prostaglandin E_2 _(PGE_2_) expression were measured by ELISA. Effects of curcumin and resveratrol were evaluated by treating TNFα-stimulated adipocytes with each compound and 1) assessing the activation state of the NF-κB signaling pathway and 2) measuring inflammatory gene expression by qRT-PCR and ELISA.

**Results:**

Both preadipocytes and differentiated adipocytes express the genes for TNF-α, IL-6, and COX-2, key mediators of the inflammatory response. Preadipocytes were also found to express IL-1β; however, IL-1β expression was absent in differentiated adipocytes. TNF-α treatment activated NF-κB signaling in differentiated adipocytes by inducing IκB degradation and NF-κB translocation to the nucleus, and as a result increased IL-6 (6-fold) and COX-2 (2.5-fold) mRNA levels. TNF-α also activated IL-1β gene expression in differentiated adipocytes, but had no effect on endogenous TNF-α mRNA levels. No detectable TNFα or IL-1β was secreted by adipocytes. Curcumin and resveratrol treatment inhibited NF-κB activation and resulted in a reduction of TNF-α, IL-1β, IL-6, and COX-2 gene expression (IC_50 _= 2 μM) and a reduction of secreted IL-6 and PGE_2 _(IC_50 _~ 20 μM).

**Conclusion:**

Curcumin and resveratrol are able to inhibit TNFα-activated NF-κB signaling in adipocytes and as a result significantly reduce cytokine expression. These data suggest that curcumin and resveratrol may provide a novel and safe approach to reduce or inhibit the chronic inflammatory properties of adipose tissue.

## Background

Obesity is now known to play a causal role in the complex disease state of metabolic syndrome, as well as being a significant risk factor for cardiovascular disorders and diabetes [1,2]. Although once thought to serve as a simple storage depot for excess fats, adipose tissue also regulates organismic metabolism through a variety of signaling mechanisms including autonomic nervous stimulation and secreted hormones [3,4]. When in proper balance, these regulatory mechanisms effectively control energy preservation (lipogenesis) during the post-prandial period and energy mobilization (lipolysis) during times of increased energy expenditure.

In addition to these mechanisms of metabolic regulation, adipose tissue is also capable of producing proteins that are classical mediators of the inflammatory response. In the early 1990's, it was discovered that adipocytes synthesize and secrete the pro-inflammatory cytokine, Tumor Necrosis Factor-alpha (TNF-α) [5]. Since then, it has been shown that a number of acute phase reactants and inflammatory mediators are made by adipocytes including plasminogen activator inhibitor-1, IL-1β, IL-6, IL-8, IL-10, IL-15, hepatocyte growth/scatter factor and prostaglandin E_2 _(PGE_2_) [6]. In fact, enough of these factors are secreted by adipocytes that overall systemic levels are significantly elevated in obese subjects [7] and a number of studies have now identified a direct correlation between body mass index (BMI) and systemic levels of inflammatory proteins [8]. These clinical observations provide key evidence linking obesity with cardiovascular disorders and begin to shed light on how low-level, chronic inflammation adversely affects cardiovascular function in obese subjects.

Recent evidence suggests that cytokine expression in adipose tissue is initiated by crosstalk occurring between adipocytes and macrophages [8-11]. Macrophages typically account for 5–10% of cells within adipose tissue obtained from non-obese donors; however, in diet-induced obesity, macrophage infiltration can account for up to 60% of all cells in adipose tissue [12]. Cytokines secreted by macrophages, including TNFα, IL-1β and IL-6, are known to stimulate cytokine expression in adipocytes [13-15] and establish a paracrine loop between these two cell types [16]. This paracrine stimulation in turn elevates systemic cytokine levels observed in obese individuals. In *bone fide *inflammatory cells, cytokine gene expression is activated following activation of the Nuclear Factor-kappaB (NF-κB) signal transduction pathway [17]. Activation of the NF-κB pathway is mediated by a variety of signals including those initiated from the TNFα receptor and Toll-like receptor family. NF-κB itself is a heterodimeric transcription factor that is retained in the cytosol in its inactive state by complexing with a set of inhibitory proteins designated IκB. Upon receptor activation of NF-κB signaling the IκB complex is phosphorylated by IκB kinase (IKK). This in turn leads to its dissociation from NF-κB and rapid degradation by the proteosome. Free NF-κB is then able to translocate to the nucleus where it binds to specific promoter elements resulting in the activation of a battery of genes, including those encoding for inflammatory proteins.

In adipocytes, both expression and activity of NF-κB increase during differentiation [18] suggesting that it is a key player in mediating adipose-specific cytokine expression. Moreover, excessive NF-κB activity has been associated with the development of type 2 diabetes as obese individuals have high circulating levels of TNF-α, IL-1β and IL-6 that, like cardiovascular risk, directly correlate with insulin resistance [7,19,20]. Collectively, these observations suggest that therapeutic targeting of the NF-κB signaling pathway in adipose tissue represents a logical pursuit to reduce systemic cytokine levels and reverse their negative influence on cardiovascular function and diabetic progression.

There are no shortages of reported inhibitors of NF-κB activation as they now number in the hundreds [21]. Unfortunately, the targets for many of these inhibitors are not necessarily restricted to the NF-κB pathway raising concerns of potential side effects due to off-target inhibition. As an alternative, we have investigated the effectiveness of certain natural products to inhibit the NF-κB signaling pathway [22]. Many natural products have been shown to possess low level toxicity and potent anti-inflammatory properties by targeting similar pathways as non-steroidal anti-inflammatory drugs (NSAIDs). Two natural polyphenols of particular interest are curcumin and resveratrol. These natural products are modest inhibitors of NF-κB activation and inflammatory gene expression [22-30] and have proven safe in human clinical trials [31-33]. In the present study, we examined if curcumin and resveratrol are also able to inhibit NF-κB activation in adipocytes and in doing so inhibit cytokine expression in these cells. We believe that using natural products to inhibit the chronic inflammatory response of adipose tissue may provide a novel approach to reduce systemic cytokine levels which in turn is expected to improve cardiovascular health and insulin sensitivity.

## Methods

### Reagents

TNFα was obtained by R&D Systems, Minneapolis, MN and was used in all experiments at a final concentration of 20 ng/ml. Lipopolysaccharide (LPS) was purchased from Sigma, St. Louis, MO and used to activate BV-2 murine macrophages at a final concentration of 20 μg/ml. Curcumin was synthesized in the lab [34] and resveratrol was purchased from A.G. Scientific Inc., San Diego, CA.

### Cell culture and adipocyte differentiation

For our studies, we utilize an *in vitro *cell culture system that has been extensively characterized for adipocyte differentiation, namely mouse 3T3-L1 fibroblasts [35]. Following induction into the differentiation pathway, 3T3-L1 cells undergo growth arrest, become spherical, and form large intracellular lipid droplets. Subcutaneous implantation of these cells in mice results in tissue masses that are histologically indistinguishable from white adipose tissue [36,37]. 3T3-L1 cells were obtained from American Type Culture Collection (Manassas, VA) and grown in Dulbecco's modified Eagle's medium (DMEM) (Invitrogen, Carlsbad, CA) supplemented with 10% (v/v) fetal calf serum (Irvine Scientific, Santa Ana, CA), 1 mM sodium pyruvate, 0.1 mM non-essential amino acids, 2 mM L-glutamine, 100 μg/ml streptomycin sulfate, and 100 units/ml penicillin. Cells were cultured at 37°C with 10% CO_2 _and passaged twice weekly. To differentiate 3T3-L1 cells into adipocytes, cells were incubated with 250 nM dexamethasone, 450 μM 3-isobutyl-1-methylxanthine, and 167 nM insulin for 2 days, followed by 167 nM insulin for an additional 3 days.

BV-2 murine macrophages (a gift from Dr. Paul Stemmer, Wayne State University, Detroit, MI) were grown in RPMI-1640 (Hyclone^®^, Logan, UT) supplemented with 10% (v/v) fetal calf serum, 1 mM sodium pyruvate, 2 mM L-glutamine, 100 μg/ml streptomycin sulfate, and 100 units/ml penicillin. Cells were cultured at 37°C with 5% CO_2 _and passaged twice weekly.

### qRT-PCR and RT-PCR analysis

Total RNA was purified from cells using RNeasy (Qiagen, Valencia, CA) and converted to cDNA using TaqMan^® ^Reverse Transcriptase (Applied Biosystems, Branchburg, NJ). Cyclooxygenase-2 (COX-2), IL-1β, IL-6, TNFα, and β-actin expression levels were measured by quantitative Real-Time PCR analysis (qRT-PCR) of cDNA samples. Gene and primer information can be found in Table 1. Amplification of leptin and macrophage specific markers F4/80 and Mac-1 was performed by reverse transciptase-PCR (RT-PCR).

**Table 1:** Gene and primer information used in this study.

**Gene**	**GenBank no**.	**5' primer – top sequence** **3' primer – bottom sequence**	**Intron flanked**	**Base pairs amplified**
COX-2	NM_011198	TGGGGTGATGAGCAACTATT AAGGAGCTCTGGGTCAAACT	7	132
IL-1β	NM_008361	GACCTTCCAGGATGAGGACA AGCTCATATGGGTCCGACAG	3	183
IL-6	NM_031168	AGTTGCCTTCTTGGGACTGA CAGAATTGCCATTGCACAAC	2	191
TNFα	NM_013693	ACGGCATGGATCTCAAAGAC GTGGGTGAGGAGCACGTAGT	none	116
β-actin	NM_007393	CCTGAACCCTAAGGCCAACC CAGCTGTGGTGGTGAAGCTG	3	287
leptin	NM_008493	TGACACCAAAACCCTCATCA TCATTGGCTATCTGCAGCAC	2	213
F4/80	NM_010130	GCTGTGAGATTGTGGAAGCA CTGTACCCACATGGCTGATG	15	135
Mac-1	NM_008401	AAGGATTCAGCAAGCCAGAA TAGCAGGAAAGATGGGATGG	20	136

qRT-PCR was performed using ABsolute QPCR SYBR Green Mix (Fisher Scientific, Atlanta, GA) with the following cycling parameters: 1 cycle, 95°C, 15 min; 40 cycles, 95°C, 15 sec, 63°C, 1 min. Changes in gene expression were determined by the Comparative C_T _method. Since β-actin gene expression is unaffected by TNFα treatment, β-actin mRNA levels were quantified in each sample using identical cycling conditions and used to normalize values obtained for COX-2, IL-1β, IL-6, and TNFα expression. Amplified products were separated on 3% agarose gels and stained with Gel Star^® ^(Cambrex, Rockland, ME).

### Immunoblotting

Cell lysates were prepared using 1× Laemmli sample buffer (Sigma-Aldrich). After heating samples at 95°C for 10 min, they were vortexed on high for 20 s to shear DNA and reduce viscosity. Proteins were then separated by SDS-PAGE and transferred to PVDF membrane (0.2 μm, BioRad, Hercules, CA) using a wet tank transfer system (BioRad). Membranes were blocked with 20 mM Tris, pH 7.4, 150 mM NaCl (TBS) containing 0.1% (v/v) Tween-20, 5% (v/v) calf serum for 30 minutes at 23°C and incubated with either anti-IκB monoclonal antibody (2 μg/ml, Imgenex, San Diego, CA) or anti-β-actin monoclonal antibody (1:500, no. A-4700, Sigma-Aldrich) for 24 h at 23°C. Membranes were washed three times (10 min each) with TBS, 0.1% (v/v) Tween-20, and bound antibodies were detected with goat anti-mouse HRP-conjugated secondary antibody (1:3000, BioRad) followed by chemiluminescence detection with Immobilon™ Western according to the manufacturer's instructions (Millipore, Billerica, MA). Images were captured using a Syngene GeneGnome system equipped with a Peltier-cooled 16-bit CCD camera and saturation detection. Densitometry was performed using ImageJ software (version 1.37; National Institutes of Health, ).

### NF-κB nuclear localization assay

BV-2 murine macrophages were cultured in the absence or presence of LPS (20 μg/ml) for 24 h. 3T3-L1-derived adipocytes were cultured in the absence or presence of TNFα (20 ng/ml) or incubated with TNFα together with curcumin or resveratrol or vehicle alone (dimethylsulfoxide at 0.1% final concentration) for 62 h. Nuclear localized NF-κB was quantified using a Transcription Factor ELISA Kit to detect activated p65 subunit of NF-κB (Panomics, Fremont, CA). All reagents required for preparing nuclear extracts and performing ELISA assays were included and their use was described by the manufacturer.

### Cytotoxicity assay

Cells were grown in 96-well plates to 80–90% confluency. Media was replaced with fresh complete media containing the indicated concentrations of curcumin or resveratrol, or vehicle alone (dimethylsulfoxide at 0.1% final concentration). After a 24 h incubation, WST-1 (Roche Molecular Biochemicals, Indianapolis, IN) was added to the cultures to a final concentration of 10% (vol/vol). Following an additional incubation at 37°C for 60 min, absorbance was recorded for each well (450 nm; reference wavelength, 690 nm).

### Cytokine and PGE_2 _ELISA

Quantitation of cytokine protein levels from cell culture supernatants was done by ELISA Ready-SET-Go! kit (eBioscience, San Diego, CA) per manufacturer's instructions. Parameter™ PGE_2 _competitive binding ELISA kit (R&D systems, Minneapolis, MN) was used to measure PGE_2 _levels.

### Statistical analyses

All experimental protocols were done in at least triplicate points and error bars represent standard deviations of mean values. Student's t-test was performed on some figures using data sets composed of a minimum of triplicate values. Comparison of data sets resulting in p values < 0.05 were considered statistically significant.

## Results

### Cytokine expression profile in 3T3-L1 preadipocytes and differentiated adipocytes

**Figure 1:**
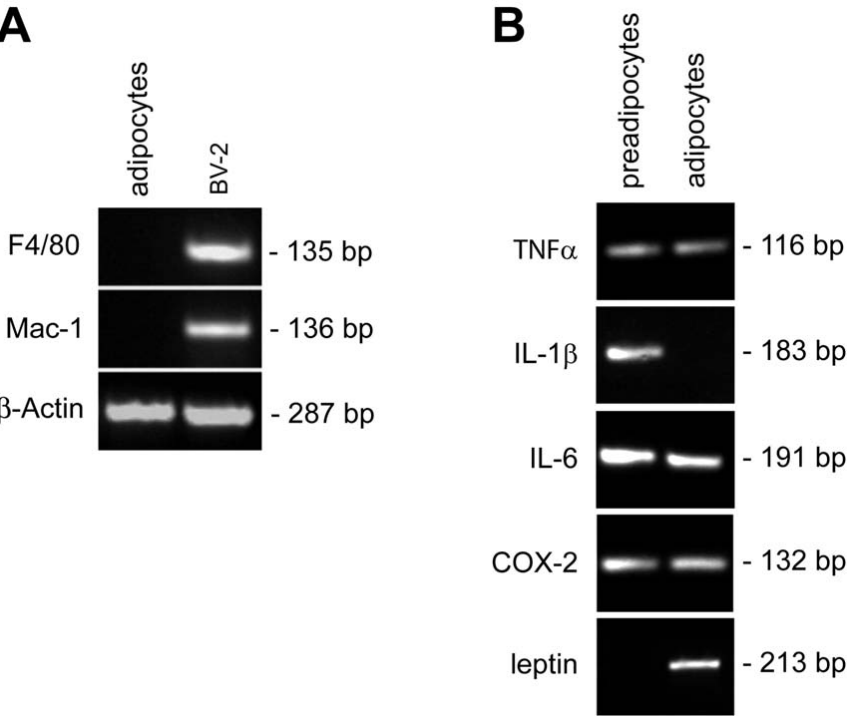
**Cytokine and COX-2 expression profile in 3T3-L1 preadipocytes and differentiated adipocytes**. (A) 3T3-L1 cells were incubated with adipocyte differentiation reagents (250 nM dexamethasone, 450 μM 3-isobutyl-1-methylxanthine, and 167 nM insulin) for two days followed by incubation with insulin (167 nM) for an additional three days. Total RNA was extracted from 3T3-L1-derived adipocytes and BV-2 murine macrophages, converted to cDNA and subjected to PCR analysis using primers specific for Mac-1 and F4/80. β-actin amplification was performed in parallel as a loading control. (B) 3T3-L1 cells were cultured without (preadipocytes) or with (adipocytes) differentiation reagents as described for (A). Total RNA was extracted from cells and processed for PCR analysis using primers for the indicated genes. Amplification of leptin sequence confirmed differentiation of preadipocytes.

Immunocytochemical analysis has shown that preadipocytes often express macrophage specific antigens [38] suggesting that preadipocytes are derived from a monocytic cell lineage. Because our studies are focused on cytokine expression by adipocytes, we first needed to confirm that our mature, fully differentiated 3T3-L1-derived adipocytes have not undergone conversion to a macrophage line. We addressed this need by determining if our cultured adipocytes express the macrophage-specific markers, Mac-1 and F4/80. Rather than assessing if these markers are expressed by the more common method of flow cytometry, we chose to test for expression by performing the more sensitive reverse transcriptase-PCR analysis. As expected, both markers were expressed in our positive control cell line, BV-2 murine macrophages (Fig. 1A). By contrast, no expression could be detected in our 3T3-L1-derived adipocytes, confirming that differentiating 3T3-L1 cells does not result in conversion to a macrophage-like phenotype.

With this criteria met, we next examined the cytokine expression profile of 3T3-L1 preadipocytes as well as cytokine expression after differentiation to adipocytes. Specifically, we assessed TNFα, IL-1β, IL-6, and COX-2 expression using RT-PCR analysis. We included COX-2 in our list of inflammatory genes to examine since its expression, like the other inflammatory mediators, has also been associated with obesity [39]. As shown in Figure 1B, both 3T3-L1 preadipocytes and differentiated adipocytes express several key mediators of the inflammatory response: TNFα, IL-6, and COX-2. Notably, preadipocytes were found to express IL-1β; however, IL-1β expression was absent in fully differentiated adipocytes.

### TNFα treatment activates IL-1β expression in differentiated adipocytes and increases expression of IL-6 and COX-2

**Figure 2:**
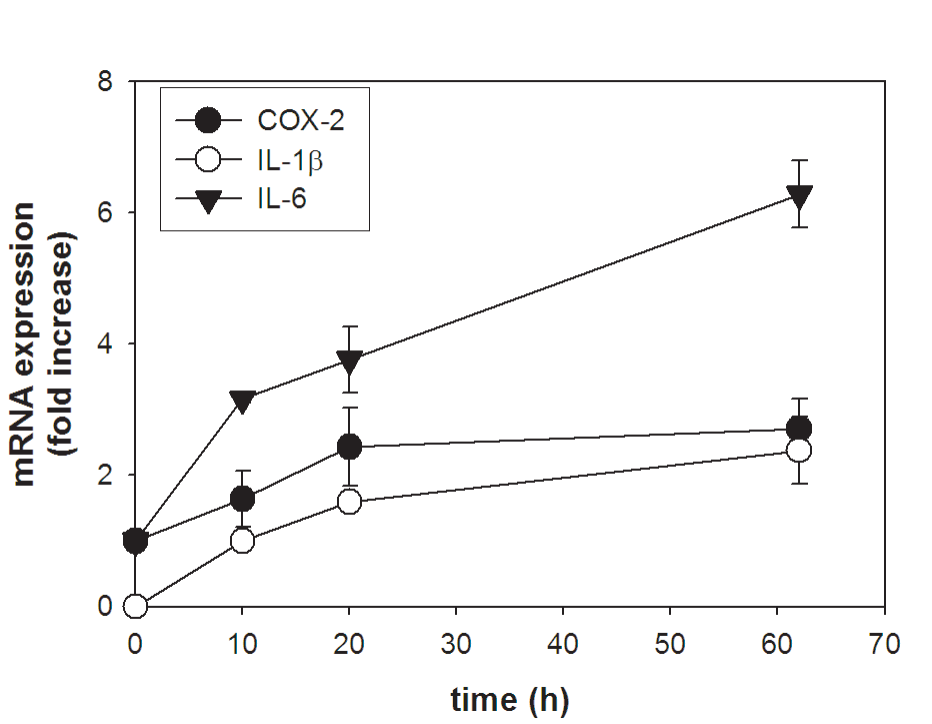
**TNFα treatment increases expression of IL-1β, IL-6 and COX-2 expression in differentiated adipocytes**. 3T3-L1 preadipocytes were differentiated into adipocytes as in Fig. 1. Following differentiation, cells were incubated with TNFα for the indicated times. Total RNA was extracted and gene expression levels were measured by qRT-PCR using the Comparative C_T _method. Amplification of β-actin sequence was performed in parallel and used to normalize values obtained for target genes. Expression levels for IL-6 and COX-2 were determined by comparing values obtained at each time point following TNFα stimulation to values obtained from untreated cells. Expression of these genes in untreated adipocytes was assigned a value of 1 to determine fold-changes in expression due to TNFα stimulation. Expression of IL-1β resulting from TNFα stimulation was assessed by comparing later time points (20 and 62 h) to 10 h TNFα treatment time since no IL-1β expression could be measured in untreated adipocytes. For this analysis, IL-1β expression measured at 10 h post-TNFα treatment was assigned a value of 1 to determine fold-changes in expression at later time points.

Infiltrating macrophages are the major source of TNFα within adipose tissue [6]. Since TNFα is thought to initiate the paracrine crosstalk between macrophages and adipocytes [9,16,40], we examined if TNFα stimulation is able to alter TNFα, IL-1β, IL-6, and COX-2 gene expression in differentiated adipocytes. Differentiated adipocytes were incubated with TNFα and target gene expression was measured by qRT-PCR. We found that TNFα treatment of differentiated adipocytes did indeed increase IL-6 and COX-2 expression in a time dependent manner (Fig. 2). After 62 h of TNFα incubation, IL-6 gene expression was elevated by 6-fold and COX-2 expression was increased by 2.5-fold. Moreover, TNFα treatment also activated IL-1β gene expression in adipocytes and increased its levels by ~2-fold after 62 h treatment. TNFα treatment had no measurable effect on TNFα gene expression in differentiated adipocytes (data not shown).

### Activation of NF-κB in adipocytes

**Figure 3:**
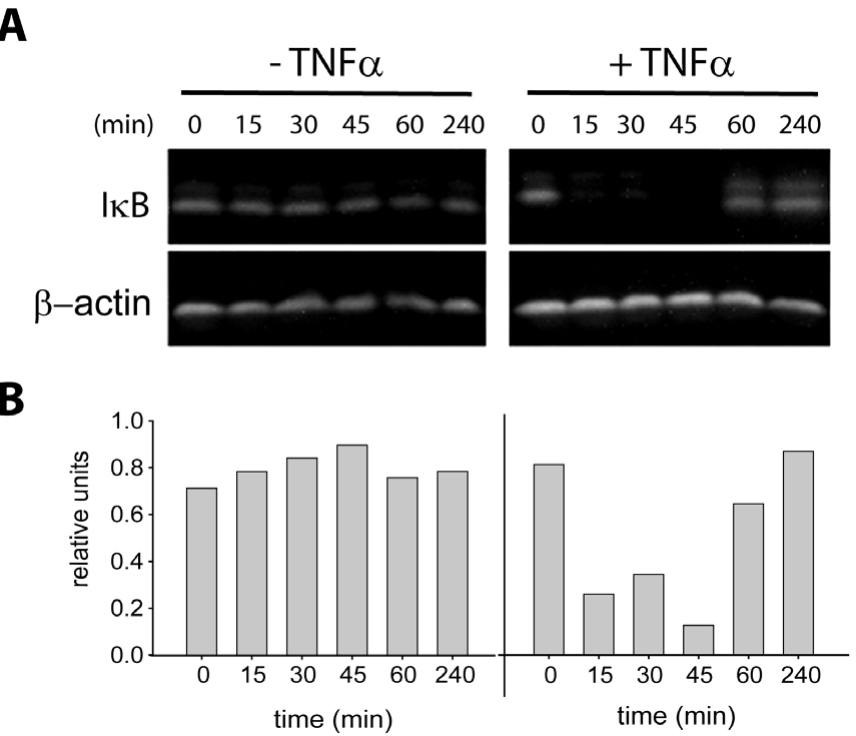
**TNFα treatment promotes degradation of IκB in adipocytes**. (A) Adipocytes were incubated without (left panels) or with (right panels) TNFα for the indicated times. Total cellular proteins were solubilized and subject to immunoblot analysis using anti-IκB monoclonal antibody (upper panels). Samples were also immunoblotted with anti-β-actin monoclonal antibodies in parallel as a loading control (lower panels). (B) Densitometric quantitation of IκB shown in (A).

To further examine the NF-κB signaling pathway in adipocytes, we investigated the immediate upstream events that are responsible for NF-κB activation and its translocation to the nucleus. Activation of NF-κB in inflammatory cells, and in many other cell types, is known to require its dissociation from its inhibitory subunit, IκB. This dissociation occurs following phosphorylation of IκB and subsequent IκB degradation by the proteosome. To determine if this same activation sequence occurs in adipocytes, we incubated cells with TNFα and examined if IκB is degraded as a result of this stimulus. In the absence of TNFα, no evidence of IκB degradation was found in adipocytes (Fig. 3). With TNFα treatment, we found that IκB was rapidly degraded within 15 minutes and reappeared after 60 minutes. This time course is similar to that found for inflammatory cells [41], suggesting that the upstream signaling events responsible for IκB phosphorylation and degradation are comparable between inflammatory cells and adipocytes.

**Figure 4:**
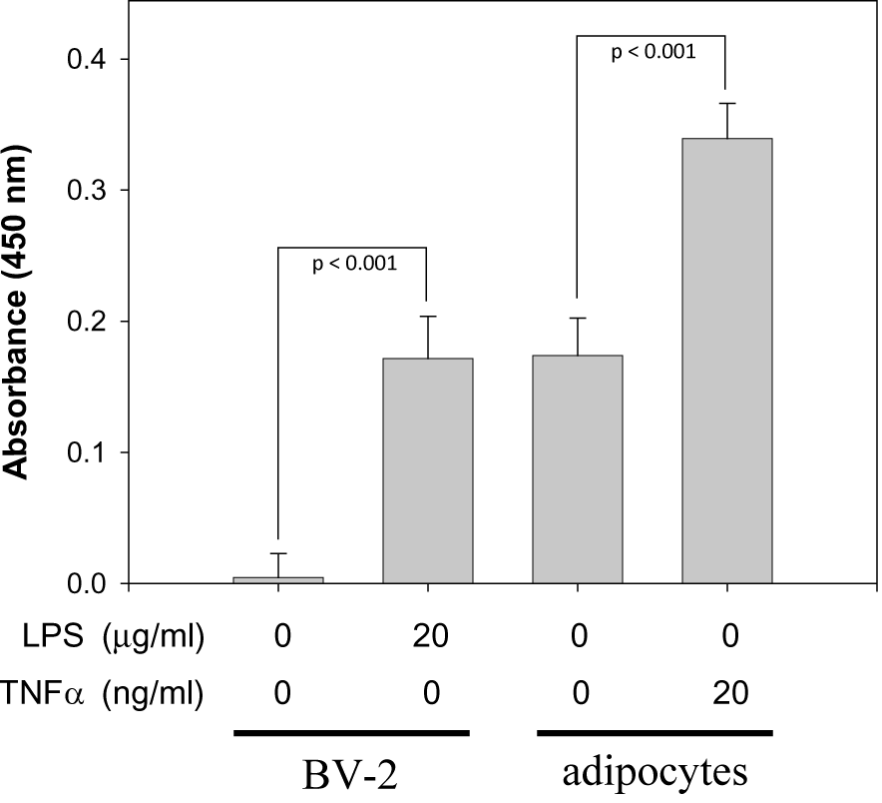
**TNFα treatment increases NF-κB translocation to the nucleus in adipocytes**. BV-2 murine macrophages or adipocytes were incubated without or with LPS or TNFα for 24 h and 62 h, respectively. Nuclear extracts were prepared and NF-κB levels were quantified by ELISA analysis using a commercially available system. Student's t-test was performed to determine statistical probability (p).

We also examined the effect of TNFα treatment on the activation state of NF-κB in adipocytes. Since NF-κB activation is accompanied by its translocation to the nucleus, we compared levels of NF-κB found in nuclear extracts from TNFα-treated and untreated cells. Using a commercially available ELISA, we found, as expected, lipopolysaccharide (LPS) treatment of murine macrophages induced the translocation of NF-κB into the nucleus (Fig. 4). Interestingly, untreated adipocytes demonstrate some NF-κB in nuclear extracts. TNFα-stimulation increased the amount of NF-κB translocation by ~2-fold, which corroborates data presented in Figure 3 showing TNFα-mediated IκB degradation.

### Curcumin and resveratrol show very little cytotoxicity to adipocytes

**Figure 5:**
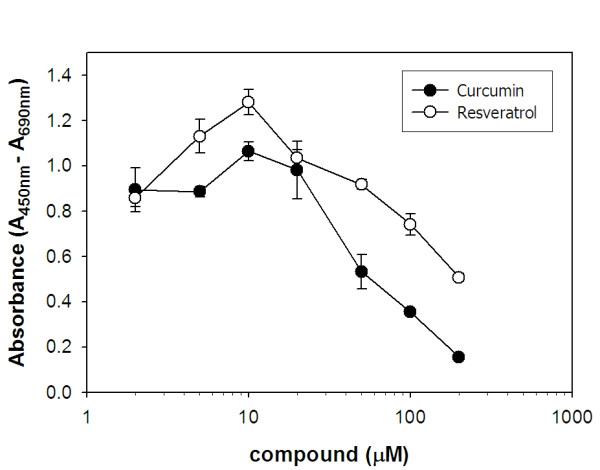
**Curcumin and resveratrol show cytotoxicity in adipocytes only at relatively high concentrations**. Adipocytes were cultured without or with the indicated concentrations of curcumin (●) or resveratrol (○) in complete media containing 10% fetal bovine serum for 24 h. Wst-1 reagent was then added to cultures to a final dilution of 1:10, cultures were incubated for an additional one hour and color was measured by spectroscopy (450 nm; reference wavelength, 690 nm).

An important consideration in our exploration of the therapeutic effects of curcumin and resveratrol is their low level cytotoxicities. In the drug development pipeline, many synthetic compounds fail early in the evaluation process because of high cytotoxic levels. Prior to examining if curcumin and resveratrol have an effect on cytokine production by adipocytes, we wish to confirm that these natural products are not harmful to this cell type. Although many previous studies have shown that these compounds are not cytotoxic at their effective doses, no data is available regarding their effect on adipocyte cell health. To address this issue, we incubated adipocytes with various concentrations of curcumin or resveratrol and quantified the metabolic state of cells as a measure of their viability. Consistent with data obtained for other cell types, curcumin and resveratrol demonstrate cytotoxicity only at relatively high concentrations. Extrapolation of the data shown in Figure 5 reveals LD_50 _values of 70 and 220 μM for curcumin and resveratrol, respectively.

### Curcumin and resveratrol inhibit IκB degradation and NF-κB translocation to the nucleus in adipocytes

**Figure 6:**
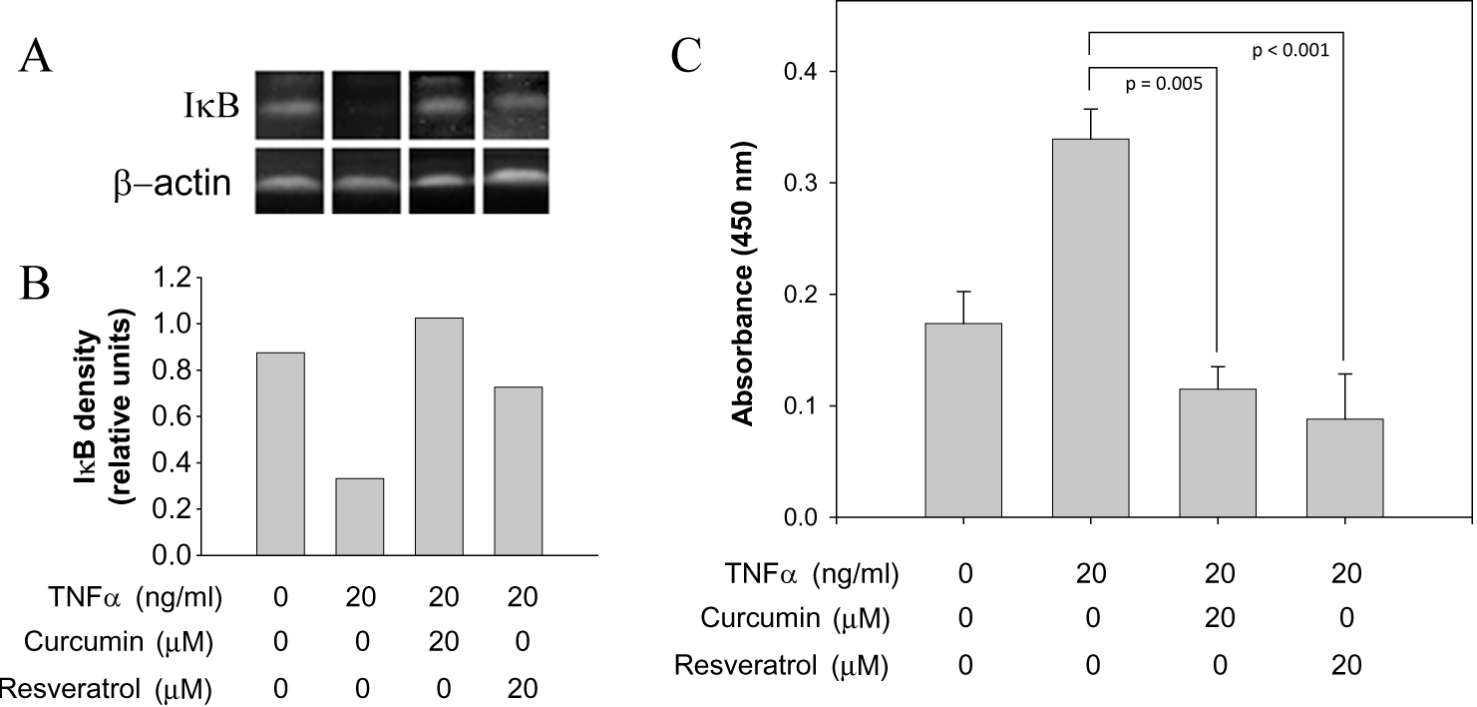
**Curcumin and resveratrol prevent IκB degradation and reduce NF-κB nuclear translocation in adipocytes**. (A) Adipocytes were incubated without or with TNFα for 20 min. In parallel, cells were also treated with TNFα and co-incubated with curcumin or resveratrol at the indicated concentrations. Total cellular proteins were solubilized and processed for immunoblot analysis using anti-IκB monoclonal antibody (upper panel) or with anti-β-actin monoclonal antibodies (lower panel). (B) Densitometric quantitation of IκB shown in (A). (C) Adipocytes were incubated without or with TNFα for 62 h. In parallel, cells were also treated with TNFα and co-incubated with curcumin or resveratrol. Nuclear extracts were prepared and NF-κB levels were quantified by ELISA analysis as in Fig. 4.

Inhibition of NF-κB activation by curcumin and resveratrol has been demonstrated in many cell types [27,42,43]; however, the effect of these natural products on NF-κB activation and cytokine production in adipocytes has not been examined. To determine if these natural products might be useful compounds in limiting NF-κB activation in adipocytes, we incubated cells with TNFα in the absence or presence of 20 μM curcumin or resveratrol and examined the effect of this treatment on IκB degradation and NF-κB translocation to the nucleus. Consistent with our results shown in Figures 3 and 4, TNFα treatment alone resulted in degradation of IκB (Fig. 6A and 6B) and increase levels of NF-κB in nuclear extracts (Fig. 6C). When cells were co-incubated with TNFα and either curcumin or resveratrol, degradation of IκB was inhibited, as was NF-κB nuclear translocation, indicating that these compounds are effective inhibitors of NF-κB activation in adipocytes.

### Curcumin and resveratrol reduce cytokine and COX-2 gene expression in adipocytes

**Figure 7:**
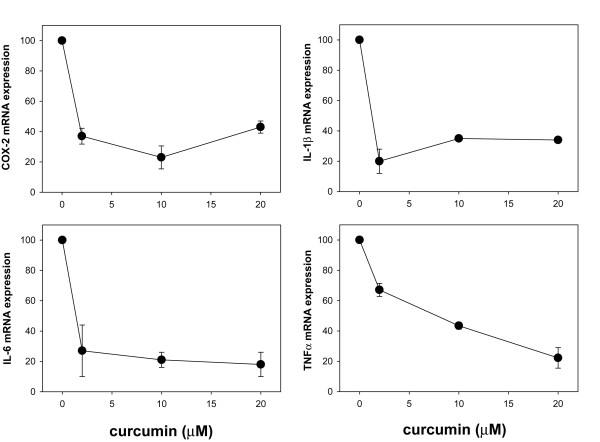
**Curcumin reduces TNFα, IL-1β, IL-6, COX-2 gene expression in differentiated adipocytes**. For IL-1β, IL-6, and COX-2 expression analysis, differentiated adipocytes were incubated with TNFα and the indicated concentrations of curcumin for 62 h. Total RNA was extracted and gene expression levels were measured by qRT-PCR using the Comparative C_T _method. For TNFα expression analysis, differentiated adipocytes were incubated with curcumin in the absence of TNFα stimulation since we found that this stimulus has no effect on TNFα expression by adipocytes. For IL-1β, IL-6, and COX-2, relative expression levels were determined by comparing values obtained from TNFα-stimulated, curcumin-treated adipocytes to TNFα-stimulated, untreated adipocytes (assigned as 100% mRNA expression). Relative expression levels of TNFα were determined by comparing values obtained from curcumin-treated adipocytes to untreated adipocytes (assigned as 100% mRNA expression).

**Figure 8:**
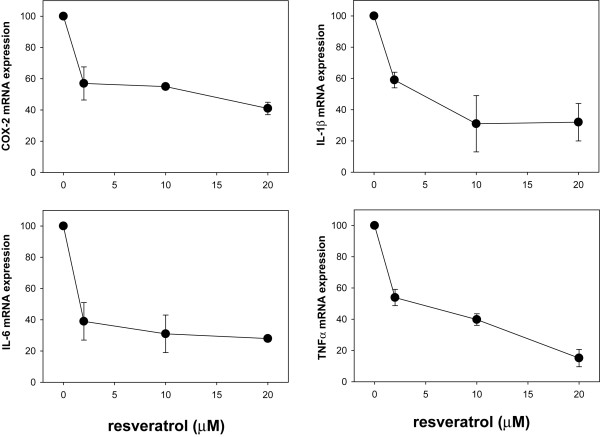
**Resveratrol reduces TNFα, IL-1β, IL-6, COX-2 gene expression in differentiated adipocytes**. Experimental method for Fig. 8 was identical to that described for Fig. 7 except that resveratrol was substituted for curcumin.

Based on these observations, we next asked if these natural products were also capable of inhibiting cytokine and COX-2 gene expression in adipocytes, thereby establishing their potential as therapeutics to counter the inflammatory response of adipose tissue. Toward this goal, we activated the NF-κB pathway in adipocytes with TNFα and incubated cells with vehicle alone or varying concentrations of curcumin or resveratrol for 62 h followed by quantification of TNFα, IL-1β, IL-6, and COX-2 gene expression by qRT-PCR analysis. We found that both curcumin (Fig. 7) and resveratrol (Fig. 8) were able to reduce expression of all four genes in a dose-dependent manner. IC_50 _values were estimated to be < 2 μM for inhibition of COX-2, IL-1β and IL-6 gene expression, and ~8 μM for inhibition of TNFα gene expression.

### Secreted cytokine protein and PGE_2 _levels are reduced by curcumin and resveratrol

**Figure 9:**
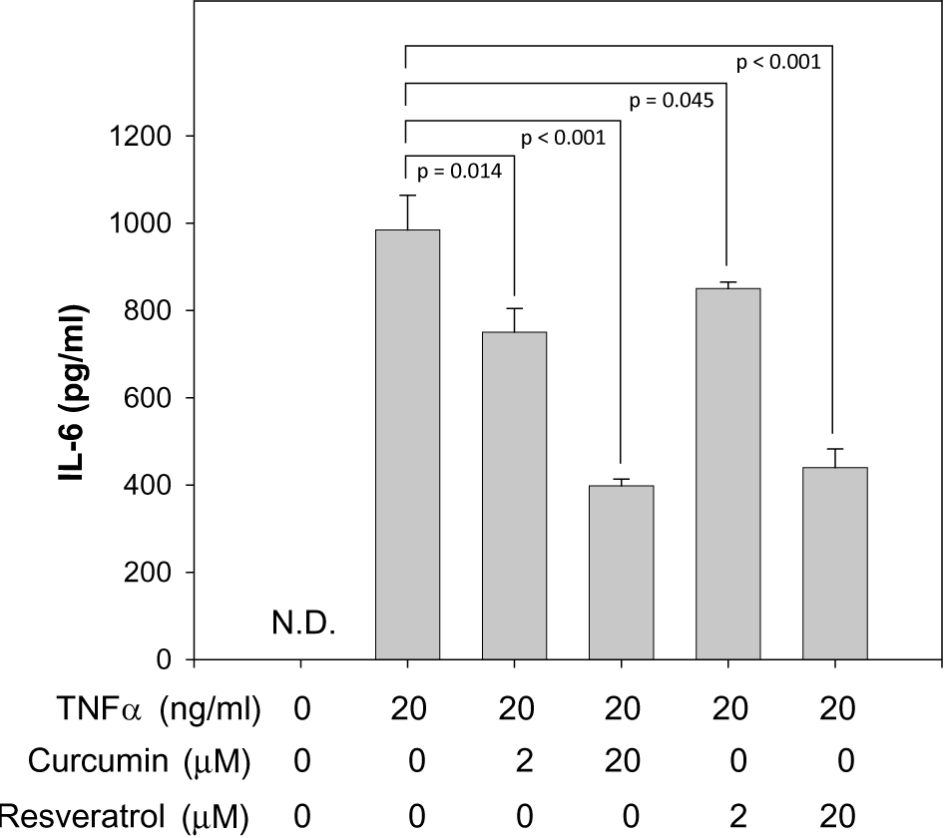
**Curcumin and resveratrol reduce secreted IL-6 levels by TNFα-stimulated adipocytes**. Adipocytes were incubated without or with TNFα. In parallel, adipocytes were also co-incubated with TNFα and 2 or 20 μM curcumin or resveratrol as indicated. After 62 h incubation, secreted levels of IL-6 were quantified by capture ELISA. Student's t-test was performed to determine statistical probability (p). N.D., none detected.

**Figure 10:**
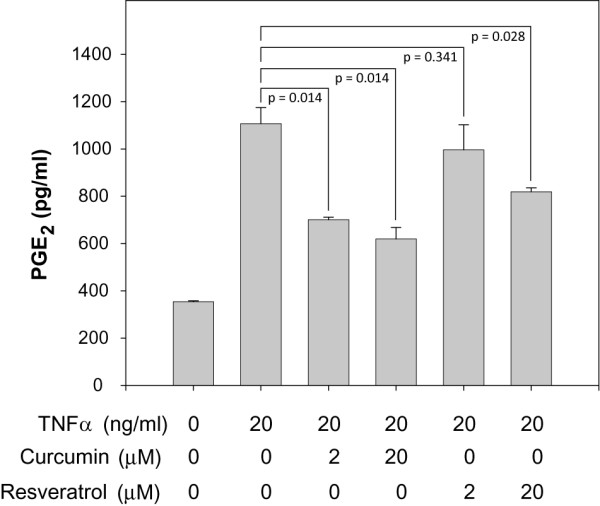
**Curcumin and resveratrol reduce secreted PGE_2 _levels by TNFα-stimulated adipocytes**. Experimental method for Fig. 10 was identical to that described for Fig. 9 except that PGE_2 _levels in culture media were quantified by competitive binding ELISA. Student's t-test was performed to determine statistical probability (p).

To extend our observations defining the effects of curcumin and resveratrol on expression of inflammatory mediators by adipocytes, we next performed ELISA-based assays to quantify their impact on secreted levels of cytokine protein and PGE_2_. PGE_2 _levels were measured since its synthesis is mediated by COX-2 and is therefore a direct reflection of COX-2 protein expression. To our surprise, we were unable to detect any TNFα or IL-1β secreted by adipocytes in spite of the presence of measurable gene expression levels (data not shown). Conversely, TNFα treatment of adipocytes significantly elevated secreted levels of IL-6 (Fig. 9) and PGE_2 _(Fig. 10). By incubating adipocytes with TNFα together with either curcumin or resveratrol, we were able to measure a dose-dependent reduction in secreted levels of IL-6 (Fig. 9) and PGE_2 _(Fig. 10).

## Conclusion

Increased adiposity is now a well established risk factor for developing complications related to metabolic syndrome and type II diabetes mellitus. Mounting evidence indicates that low level, chronic inflammation resulting from cytokines secreted by adipose tissue may play a significant role in causing, or at the very least aggravating, the inflammatory component of cardiovascular disease and in desensitizing cells to insulin leading to high circulating glucose levels. These observations suggest a hypothesis that reducing or preventing the inflammatory properties of adipose tissue represents a novel and promising therapeutic approach to curb the progression of cardiovascular disease and to restore insulin sensitivity in type II diabetics.

Macrophage infiltration has recently been postulated to be a primary stimulus for fueling the inflammatory properties of adipose tissue [44]. Monocyte chemoattractants [9,11], such as monocyte chemoattractant protein-1 (MCP-1) which is synthesized and secreted by adipocytes [45], are thought to mediate macrophage infiltration and intensify macrophage expression of TNFα [8]. TNFα has pleiotropic effects on adipocyte physiology including an induction of lipolysis to increase the mobilization of free fatty acids [46,47], activating cytokine expression [8] and promoting insulin resistance [5,48]. Observations such as these provide sufficient evidence suggesting that TNFα is the predominant factor that mediates the crosstalk between macrophages and adipocytes and that elevated TNFα levels found in obese individuals establishes a paracrine loop in adipose tissue [16] that is responsible for the elevated systemic levels of cytokines seen in obesity.

TNFα mediates its affects on adipocytes by activating the NF-κB signaling pathway [11,18]; a signaling event that has been studied extensively in the innate immune response. In conventional immune cells, activation of the NF-κB signaling pathway requires relocation of the NF-κB heterodimer from the cytoplasm to the nucleus where it functions as part of a multi-protein transcription complex controlling the expression of most inflammatory mediators. In adipose tissue, low level NF-κB activation has been identified in vivo [49] suggesting that, like in conventional immune cells, NF-κB is largely responsible for cytokine gene expression in adipocytes. Only recently has the role of NF-κB in adipose function come under scrutiny. Berg, et al., examined NF-κB expression and activity during adipocyte differentiation and found both parameters to be elevated in fully differentiated adipocytes [18]. Consistent with their findings, we were able to activate NF-κB signaling in differentiated adipocytes with TNFα treatment and in doing so demonstrate an increase in NF-κB nuclear translocation. However, to extend these observations we also examined the upstream signaling event that is directly responsible for NF-κB activation, namely IκB degradation. We found that IκB was rapidly degraded in adipocytes following TNFα treatment and with kinetics similar to those measured for true immune cells [41]. These data provide compelling evidence that NF-κB signaling in adipocytes shares a similar time course of activation as inflammatory cells.

Because the NF-κB signaling pathway is such a pleiotropic pro-inflammatory and pro-survival factor in a wide range of disorders, it has been an attractive target for small-molecule inhibitor development. Thus far almost 800 compounds have been reported to inhibit NF-κB activation [21]. A large fraction of these inhibitors include natural products that are capable of targeting multiple checkpoints in the NF-κB activation pathway. Of particular interest are the polyphenolic natural compounds, curcumin and resveratrol. Curcumin is derived from the spice turmeric, which comes from the root of *Curcuma longa *of the ginger family. It is an established inhibitor of NF-κB activation [29] and has recently been shown to specifically target IKK [24]. Inhibitors targeting IKK have so far proven to be the most effective compounds for preventing the activation of NF-κB [50-54] by directly preventing the phosphorylation of IκB, and as a consequence, block NF-κB translocation to the nucleus. Important for clinical drug development, curcumin has also been found safe in six human trials at oral doses up to 8 g/day administered for 3 months [31,32]. The other natural product that has been a focus of our laboratory is resveratrol [22]. A product of red grapes, resveratrol possesses multiple biological activities including anti-oxidant and anti-cancer activities, and like curcumin, is an inhibitor of NF-κB activation [55] through targeted inhibition of IKK [56]. In addition, although the extent of its bioavailability is still under investigation [57,58], resveratrol has been shown to be quite safe in preclinical trials [33]. In the present study, we examined if curcumin and resveratrol might represent promising therapeutics to combat the chronic inflammatory properties of adipose tissue by exploring their effects on NF-κB activation and inflammatory cytokine expression in adipocytes. We first identified that curcumin and resveratrol are able to inhibit NF-κB translocation to the nucleus in TNFα-stimulated adipocytes. Moreover, we also found that both natural products are able to prevent IκB degradation. These data establish that curcumin and resveratrol carry out their inhibitory functions either at the level of IκB phosphorylation by IKK or upstream from this checkpoint in the NF-κB activation pathway. We next examined the effects of curcumin and resveratrol on downstream gene regulation in adipocytes since NF-κB activation is largely responsible for mediating inflammatory gene expression in immune cells. Indeed, treatment of TNFα-stimulated adipocytes with curcumin or resveratrol resulted in a significant reduction in TNFα, IL-1β, IL-6, and COX-2 gene expression. The IC_50 _values measured for inhibition of IL-1β, IL-6, and COX-2 gene expression by either compound were found to be < 2 μM; for TNFα gene expression, the IC_50 _value was ~8 μM.

During the course of identifying inhibitors for NF-κB signaling, many studies will limit their analysis to measuring the effects of inhibitors on the transcriptional status of cytokine genes. Although these studies provide a wealth of data regarding the direct control of cytokine gene expression by the state of NF-κB activity, they fall short of identifying additional mechanisms of regulation at post-transcriptional levels. Limiting inhibitor identification to effects on transcriptional levels in bona fide immune cells may be acceptable since the NF-κB signaling pathway that mediates these immunological responses has been well studied [59]. However, because much less is known about potential multi-level regulatory elements in non-immune cells that may affect NF-κB signaling, cytokine expression analyses should include a quantitative assessment of secreted cytokines to identify possible post-transcriptional control of cytokine expression. By extending our analysis to measuring levels of secreted cytokines, we have identified unique expression patterns that may have significant impact on our understanding of adipocyte contributions to systemic inflammation. First, although adipocytes express TNFα mRNA, we were unable to measure any secreted TNFα by ELISA. This observation suggests that the major source of circulating TNFα found in obese subjects arises from adipose-infiltrating macrophages rather than adipocytes. A similar observation was made by Fain and colleagues when comparing isolated adipocytes to stromal vascular cells obtained from human adipose explants [6]. In this study the authors found significant amounts of TNFα secreted by stromal vascular cells, with little or no detectable TNFα secreted by adipocytes. One caveat of this study stems from the fact that the adipocytes were removed from the in vivo environment where they are exposed to macrophage-derived TNFα. Removal of TNFα-stimulation from the isolated adipocytes would discontinue signaling events that arguably might be necessary to sustain TNFα secretion by adipocytes. Our study clearly addresses this concern by demonstrating the lack of TNFα secretion in TNFα-stimulated adipocytes.

We also found that preadipocytes express the gene for IL-1β, yet differentiated adipocytes show no mRNA expression. Interestingly, TNFα treatment was able to re-activate IL-1β mRNA expression in differentiated adipocytes; however, in spite of this re-activation we were unable to detect any secreted IL-1β from treated adipocytes indicating that post-transcriptional mechanisms are in place to prevent expression of IL-1β protein. These observations may be interpreted based on the effects of long-term treatment of adipocytes with IL-1β. Such treatment has been shown to inhibit insulin receptor substrate -1 (IRS-1) expression [60] and activation [61] thereby inducing insulin resistance. By repressing IL-1β transcription during adipocyte differentiation, insulin responsiveness can be maintained for proper glucose homeostasis. Furthermore, because expansion of adipose tissue is accompanied by accelerated macrophage infiltration providing a substantial source of secreted TNFα, which we show can activate IL-1β gene expression, additional levels of regulation become necessary to prevent secretion of IL-1β protein by adipocytes. Collectively, these observations indicate that multiple regulatory checkpoints are in place to prevent IL-1β expression and ensure proper insulin responsiveness by adipocytes.

In contrast to the results obtained for measurements of secreted TNFα and IL-1β, TNFα-stimulation of adipocytes did have a pronounced effect on secreted levels of IL-6 and PGE_2_. We found little or no IL-6 secreted by unstimulated, fully differentiated adipocytes; however, when stimulated with TNFα, a significant level of secreted IL-6 was measured. In spite of a lack of secreted IL-6, we found that the IL-6 gene is expressed in unstimulated adipocytes and is responsive to TNFα stimulation as mRNA levels increased by 6-fold. These data indicate that TNFα stimulation of adipocytes not only increases transcriptional activity of the IL-6 gene, but also activates post-transcriptional events to produce secreted IL-6. Secreted PGE_2 _levels were also measured as a direct assessment of COX-2 activity. We found that TNFα-stimulation modestly increased COX-2 gene expression by 2-fold and increased secreted PGE_2 _by 3-fold over basal levels found in unstimulated adipocytes. Notably, both curcumin and resveratrol treatment of TNFα-stimulated adipocytes significantly reduced secreted levels of IL-6 and PGE_2 _in a dose-dependent manner. IC_50 _values for curcumin and resveratrol inhibition of IL-6 are estimated to be ~20 μM. By contrast, IC_50 _values for inhibition of PGE_2 _differ for each compound; ~2 μM for curcumin and > 20 μM for resveratrol. These IC_50 _values determined for secreted levels of IL-6 and PGE_2 _are noticeably higher than what was measured for inhibition of IL-6 and COX-2 gene expression. These differences are most likely due to previously unidentified effects of curcumin and resveratrol on post-transcriptional events and highlight the importance of measuring the final product in addition to transcriptional levels when identifying the quantitative effects of potential inhibitory compounds.

Whenever a compound is being developed as a potential therapeutic, issues involving in vivo bioavailability must be addressed. In this regard, data thus far presented on the pharmacokinetics of curcumin [62,63] and resveratrol [64] have been confusing and often times contradictory. Both polyphenols have relatively short half-lives in vivo as they are rapidly metabolized to their glucuronide and sulfated forms. These metabolites, readily found in the circulation, typically demonstrate very low cell permeability and questionable bioactivity when compared to their unmetabolized forms. In spite of these hurdles, the in vivo efficacies of curcumin and resveratrol have been reproducibly shown by numerous investigators. Many challenges lie ahead in order to systematically and quantitatively address the pharmacokinetics of these natural products. Immediate questions that need to be addressed to improve on in vivo efficacy include, 1) do the metabolites of curcumin and resveratrol have comparable bioactivity with the parent compounds, 2) does the circulating pool of metabolites represent a source of inhibitor that can be modified to their more active forms, and 3) can chemical substitutions be made to the base structures of curcumin and resveratrol making them more active and less susceptible to conjugation.

Most importantly for our hypothesis, the results presented here provide proof-of-principle evidence that use of curcumin and resveratrol represents a promising new therapeutic approach to reduce both local and systemic inflammatory contributions by adipose tissue. At present, we believe that the low μM IC_50 _values of curcumin and resveratrol together with their positive in vivo effects make these natural products excellent lead compounds to guide the development of more potent inhibitors of NF-κB activation and inflammatory gene expression. Toward this goal, we have recently developed chemical libraries of synthetic analogs based on the chemical structures of curcumin [23] and resveratrol [22]. Studies are currently underway to identify if these novel structural analogs improve upon the inhibitory properties of the parent compounds while also critically addressing the challenges of bioavailability and in vivo metabolism.

## Competing interests

The authors declare that they have no competing interests.

## Authors' contributions

AMG carried out the experiments in this study and participated in the experimental design, RAO provided the original conceptual framework for the study, assisted with the experimental design and finalized the manuscript for submission. All authors read and approved the final version.

## References

[B1] Grundy SM (2002). Obesity, metabolic syndrome, and coronary atherosclerosis. Circulation.

[B2] Krauss RM, Winston M, Fletcher RN, Grundy SM (1998). Obesity: impact of cardiovascular disease. Circulation.

[B3] Attele AS, Shi ZQ, Yuan CS (2002). Leptin, gut, and food intake. Biochemical Pharmacology.

[B4] Mohamed-Ali V, Pinkney JH, Coppack SW (1998). Adipose tissue as an endocrine and paracrine organ. Int J Obes Relat Metab Disord.

[B5] Hotamisligil GS, Shargill NS, Spiegelman BM (1993). Adipose expression of tumor necrosis factor-alpha: direct role in obesity-linked insulin resistance. Science.

[B6] Fain JN, Madan AK, Hiler ML, Cheema P, Bahouth SW (2004). Comparison of the release of adipokines by adipose tissue, adipose tissue matrix, and adipocytes from visceral and subcutaneous abdominal adipose tissues of obese humans. Endocrinology.

[B7] Cottam DR, Mattar SG, Barinas-Mitchell E, Eid G, Kuller L, Kelley DE, Schauer PR (2004). The chronic inflammatory hypothesis for the morbidity associated with morbid obesity: implications and effects of weight loss. Obes Surg.

[B8] Berg AH, Scherer PE (2005). Adipose tissue, inflammation, and cardiovascular disease. Circ Res.

[B9] Fantuzzi G (2005). Adipose tissue, adipokines, and inflammation. J Allergy Clin Immunol.

[B10] Tilg H, Moschen AR (2006). Adipocytokines: mediators linking adipose tissue, inflammation and immunity. Nat Rev Immunol.

[B11] Wellen KE, Hotamisligil GS (2003). Obesity-induced inflammatory changes in adipose tissue. J Clin Invest.

[B12] Weisberg SP, McCann D, Desai M, Rosenbaum M, Leibel RL, Ferrante AW (2003). Obesity is associated with macrophage accumulation in adipose tissue. J Clin Invest.

[B13] Permana PA, Menge C, Reaven PD (2006). Macrophage-secreted factors induce adipocyte inflammation and insulin resistance. Biochem Biophys Res Commun.

[B14] Lin Y, Lee H, Berg AH, Lisanti MP, Shapiro L, Scherer PE (2000). The lipopolysaccharide-activated toll-like receptor (TLR)-4 induces synthesis of the closely related receptor TLR-2 in adipocytes. J Biol Chem.

[B15] Rajala MW, Scherer PE (2003). Minireview: The adipocyte – at the crossroads of energy homeostasis, inflammation, and atherosclerosis. Endocrinology.

[B16] Suganami T, Nishida J, Ogawa Y (2005). A paracrine loop between adipocytes and macrophages aggravates inflammatory changes: role of free fatty acids and tumor necrosis factor alpha. Arterioscler Thromb Vasc Biol.

[B17] Andreakos E, Sacre S, Foxwell BM, Feldmann M (2005). The toll-like receptor-nuclear factor kappaB pathway in rheumatoid arthritis. Front Biosci.

[B18] Berg AH, Lin Y, Lisanti MP, Scherer PE (2004). Adipocyte differentiation induces dynamic changes in NF-kappaB expression and activity. Am J Physiol Endocrinol Metab.

[B19] Grimble RF (2002). Inflammatory status and insulin resistance. Curr Opin Clin Nutr Metab Care.

[B20] Pickup JC, Crook MA (1998). Is type II diabetes mellitus a disease of the innate immune system?. Diabetologia.

[B21] Gilmore TD, Herscovitch M (2006). Inhibitors of NF-kappaB signaling: 785 and counting. Oncogene.

[B22] Heynekamp J, Weber W, Hunsaker L, Gonzales A, Orlando R, Deck L, Jagt DV (2006). Substituted trans-Stilbenes, Including Analogs of the Natural Product Resveratrol, Inhibit the TNFα-induced Activation of Transcription Factor NF-B. J Med Chem.

[B23] Weber WM, Hunsaker LA, Roybal CN, Bobrovnikova-Marjon EV, Abcouwer SF, Royer RE, Deck LM, Jagt DL Vander (2006). Activation of NFkappaB is inhibited by curcumin and related enones. Bioorg Med Chem.

[B24] Aggarwal S, Ichikawa H, Takada Y, Sandur SK, Shishodia S, Aggarwal BB (2006). Curcumin (diferuloylmethane) down-regulates expression of cell proliferation and antiapoptotic and metastatic gene products through suppression of IkappaBalpha kinase and Akt activation. Mol Pharmacol.

[B25] Brennan P, O'Neill LA (1998). Inhibition of nuclear factor kappaB by direct modification in whole cells – mechanism of action of nordihydroguaiaritic acid, curcumin and thiol modifiers. Biochem Pharmacol.

[B26] Manna SK, Mukhopadhyay A, Aggarwal BB (2000). Resveratrol suppresses TNF-induced activation of nuclear transcription factors NF-kappa B, activator protein-1, and apoptosis: potential role of reactive oxygen intermediates and lipid peroxidation. J Immunol.

[B27] Nam NH (2006). Naturally occurring NF-kappaB inhibitors. Mini Rev Med Chem.

[B28] Singh S, Aggarwal BB (1995). Activation of transcription factor NF-kappa B is suppressed by curcumin (diferuloylmethane) [corrected]. J Biol Chem.

[B29] Singh S, Khar A (2006). Biological effects of curcumin and its role in cancer chemoprevention and therapy. Anticancer Agents Med Chem.

[B30] Surh YJ, Chun KS, Cha HH, Han SS, Keum YS, Park KK, Lee SS (2001). Molecular mechanisms underlying chemopreventive activities of anti-inflammatory phytochemicals: down-regulation of COX-2 and iNOS through suppression of NF-kappa B activation. Mutat Res.

[B31] Chainani-Wu N (2003). Safety and anti-inflammatory activity of curcumin: a component of tumeric (Curcuma longa). J Altern Complement Med.

[B32] Cheng AL, Hsu CH, Lin JK, Hsu MM, Ho YF, Shen TS, Ko JY, Lin JT, Lin BR, Ming-Shiang W, Yu HS, Jee SH, Chen GS, Chen TM, Chen CA, Lai MK, Pu YS, Pan MH, Wang YJ, Tsai CC, Hsieh CY (2001). Phase I clinical trial of curcumin, a chemopreventive agent, in patients with high-risk or pre-malignant lesions. Anticancer Res.

[B33] Aggarwal BB, Bhardwaj A, Aggarwal RS, Seeram NP, Shishodia S, Takada Y (2004). Role of resveratrol in prevention and therapy of cancer: preclinical and clinical studies. Anticancer Res.

[B34] Weber WM, Hunsaker LA, Gonzales AM, Heynekamp JJ, Orlando RA, Deck LM, Jagt DL Vander (2006). TPA-induced up-regulation of activator protein-1 can be inhibited or enhanced by analogs of the natural product curcumin. Biochem Pharmacol.

[B35] Green H, Meuth M (1974). An established pre-adipose cell line and its differentiation in culture. Cell.

[B36] Green H, Kehinde O (1979). Formation of normally differentiated subcutaneous fat pads by an established preadipose cell line. J Cell Physiol.

[B37] Vannier C, Gaillard D, Grimaldi P, Amri EZ, Djian P, Cermolacce C, Forest C, Etienne J, Negrel R, Ailhaud G (1985). Adipose conversion of ob17 cells and hormone-related events. Int J Obes.

[B38] Cousin B, Munoz O, Andre M, Fontanilles AM, Dani C, Cousin JL, Laharrague P, Casteilla L, Penicaud L (1999). A role for preadipocytes as macrophage-like cells. FASEB J.

[B39] Fain JN, Ballou LR, Bahouth SW (2001). Obesity is induced in mice heterozygous for cyclooxygenase-2. Prostaglandins Other Lipid Mediat.

[B40] Xu H, Barnes GT, Yang Q, Tan G, Yang D, Chou CJ, Sole J, Nichols A, Ross JS, Tartaglia LA, Chen H (2003). Chronic inflammation in fat plays a crucial role in the development of obesity-related insulin resistance. J Clin Invest.

[B41] Li Q, Verma IM (2002). NF-kappaB regulation in the immune system. Nat Rev Immunol.

[B42] Aggarwal BB, Shishodia S (2006). Molecular targets of dietary agents for prevention and therapy of cancer. Biochem Pharmacol.

[B43] Bremner P, Heinrich M (2002). Natural products as targeted modulators of the nuclear factor-kappaB pathway. J Pharm Pharmacol.

[B44] Cancello R, Clement K (2006). Is obesity an inflammatory illness? Role of low-grade inflammation and macrophage infiltration in human white adipose tissue. Bjog.

[B45] Christiansen T, Richelsen B, Bruun JM (2005). Monocyte chemoattractant protein-1 is produced in isolated adipocytes, associated with adiposity and reduced after weight loss in morbid obese subjects. Int J Obes (Lond).

[B46] Ryden M, Dicker A, van Harmelen V, Hauner H, Brunnberg M, Perbeck L, Lonnqvist F, Arner P (2002). Mapping of early signaling events in tumor necrosis factor-alpha – mediated lipolysis in human fat cells. J Biol Chem.

[B47] Souza SC, Palmer HJ, Kang YH, Yamamoto MT, Muliro KV, Paulson KE, Greenberg AS (2003). TNF-alpha induction of lipolysis is mediated through activation of the extracellular signal related kinase pathway in 3T3-L1 adipocytes. J Cell Biochem.

[B48] Hotamisligil GS, Murray DL, Choy LN, Spiegelman BM (1994). Tumor necrosis factor alpha inhibits signaling from the insulin receptor. Proc Natl Acad Sci USA.

[B49] Carlsen H, Moskaug JO, Fromm SH, Blomhoff R (2002). In vivo imaging of NF-kappa B activity. J Immunol.

[B50] Braun T, Carvalho G, Coquelle A, Vozenin MC, Lepelley P, Hirsch F, Kiladjian JJ, Ribrag V, Fenaux P, Kroemer G (2006). NF-kappaB constitutes a potential therapeutic target in high-risk myelodysplastic syndrome. Blood.

[B51] Braun T, Carvalho G, Fabre C, Grosjean J, Fenaux P, Kroemer G (2006). Targeting NF-kappaB in hematologic malignancies. Cell Death Differ.

[B52] Haefner B (2006). Targeting NF-kappaB in anticancer adjunctive chemotherapy. Cancer Treat Res.

[B53] Magne N, Toillon RA, Bottero V, Didelot C, Houtte PV, Gerard JP, Peyron JF (2006). NF-kappaB modulation and ionizing radiation: mechanisms and future directions for cancer treatment. Cancer Lett.

[B54] Redell MS, Tweardy DJ (2005). Targeting transcription factors for cancer therapy. Curr Pharm Des.

[B55] Kundu JK, Surh YJ (2004). Molecular basis of chemoprevention by resveratrol: NF-kappaB and AP-1 as potential targets. Mutat Res.

[B56] Kundu JK, Shin YK, Kim SH, Surh YJ (2006). Resveratrol inhibits phorbol ester-induced expression of COX-2 and activation of NF-kappaB in mouse skin by blocking IkappaB kinase activity. Carcinogenesis.

[B57] Walle T, Hsieh F, DeLegge MH, Oatis JE, Walle UK (2004). High absorption but very low bioavailability of oral resveratrol in humans. Drug Metab Dispos.

[B58] Yu C, Shin YG, Chow A, Li Y, Kosmeder JW, Lee YS, Hirschelman WH, Pezzuto JM, Mehta RG, van Breemen RB (2002). Human, rat, and mouse metabolism of resveratrol. Pharm Res.

[B59] Carmody RJ, Chen YH (2007). Nuclear factor-kappaB: activation and regulation during toll-like receptor signaling. Cell Mol Immunol.

[B60] Jager J, Gremeaux T, Cormont M, Le Marchand-Brustel Y, Tanti JF (2007). Interleukin-1beta-induced insulin resistance in adipocytes through down-regulation of insulin receptor substrate-1 expression. Endocrinology.

[B61] Lagathu C, Yvan-Charvet L, Bastard JP, Maachi M, Quignard-Boulange A, Capeau J, Caron M (2006). Long-term treatment with interleukin-1beta induces insulin resistance in murine and human adipocytes. Diabetologia.

[B62] Hsu CH, Cheng AL (2007). Clinical studies with curcumin. Adv Exp Med Biol.

[B63] Sharma RA, Steward WP, Gescher AJ (2007). Pharmacokinetics and pharmacodynamics of curcumin. Adv Exp Med Biol.

[B64] Baur JA, Sinclair DA (2006). Therapeutic potential of resveratrol: the in vivo evidence. Nat Rev Drug Discov.

